# Contrast Dependence of Smooth Pursuit Eye Movements following a Saccade to Superimposed Targets

**DOI:** 10.1371/journal.pone.0037888

**Published:** 2012-05-21

**Authors:** Mazyar Fallah, John H. Reynolds

**Affiliations:** 1 School of Kinesiology and Health Science, York University, Toronto, Ontario, Canada; 2 Systems Neurobiology Laboratory, The Salk Institute for Biological Studies, La Jolla, California, United States of America; University of Muenster, Germany

## Abstract

Dorsal stream areas provide motion information used by the oculomotor system to generate pursuit eye movements. Neurons in these areas saturate at low levels of luminance contrast. We therefore hypothesized that during the early phase of pursuit, eye velocity would exhibit an oculomotor gain function that saturates at low luminance contrast. To test this, we recorded eye movements in two macaques trained to saccade to an aperture in which a pattern of dots moved left or right. Shortly after the end of the saccade, the eyes followed the direction of motion with an oculomotor gain that increased with contrast before saturating. The addition of a second pattern of dots, moving in the opposite direction and superimposed on the first, resulted in a rightward shift of the contrast-dependent oculomotor gain function. The magnitude of this shift increased with the contrast of the second pattern of dots. Motion was nulled when the two patterns were equal in contrast. Next, we varied contrast over time. Contrast differences that disappeared before saccade onset biased post-saccadic eye movements at short latency. Changes in contrast occurring during or after saccade termination did not influence eye movements for approximately 150 ms. Earlier studies found that eye movements can be explained by a vector average computation when both targets are equal in contrast. We suggest that this averaging computation may reflect a special case of divisive normalization, yielding saturating contrast response functions that shift to the right with opposed motion, averaging motions when targets are equated in contrast.

## Introduction

When two moving targets appear in the visual field, the early phase of an ensuing pursuit eye movement can be fit by a weighted average of the motions of the two stimuli [1], [2]. This is thought to be indicative of how the motions of multiple objects are processed prior to selection mechanisms, such as attention or the pursuit system. In these studies, the moving targets were equal in size, shape, and contrast. The weighted average was based on the differing directions of motion. We sought to examine how contrast, which has previously been shown to automatically bias the selection of objects [3]–[5], interacts with motion processing. The results will constrain models of the mechanisms underlying motion processing and smooth pursuit.

We measured smooth eye movements immediately after macaques made a saccade to one or two moving stimuli, as we varied their luminance contrasts. Targets were superimposed patterns of dots within a fixed aperture, which gave rise to the percept of transparent motion. This choice of stimuli has the advantage that both stimuli appear at the location of the saccade. When only one stimulus was presented, post-saccadic eye movements were in its motion direction, with an oculomotor gain that saturated at relatively low luminance contrast. Introducing the oppositely moving stimulus caused this saturating function to shift to the right, a shift that increased monotonically with the contrast of the added stimulus, nulling the eye movement when the stimuli were equal in contrast. To characterize the time course of the onset of this contrast-dependent bias we carried out a second set of experiments in which the contrasts of the two stimuli were equal during fixation but became different during the saccade. In a second condition, the contrast difference was present during fixation but disappeared during the saccade, enabling us to examine whether the bias survives when the two stimuli shift from the periphery to the fovea during a saccade.

## Materials and Methods

### Ethics Statement

All animal experiments were reviewed and approved by Salk Institute Animal Care and Use Committee (#03–020) and performed in compliance with the Animal Welfare Act and the ILAR Guide to the Care and Use of Laboratory Animals. Primates were housed, fed and handled according to contemporary standards under the supervision of qualified laboratory animal veterinarians. Every effort was made to alleviate animal discomfort and pain by appropriate and routine use of anesthetic and/or analgesic agents. All invasive procedures were performed under general anesthesia following strict aseptic procedures. Animals were observed continuously until fully recovered and provided with adequate pre- and post-operative analgesia.

### Subjects

Two adult male monkeys (Macaca mulatta) were each implanted with a head holding device.

### Stimuli and Task

**Figure 1:**
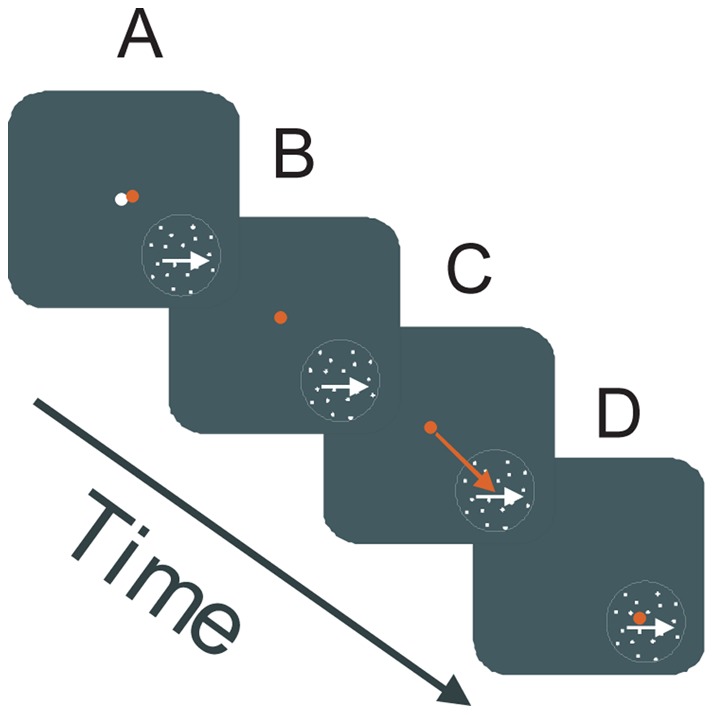
Task Design. A. Monkeys fixated a central fixation point (white dot) and a translating pattern of dots appeared in the lower visual field, either to the left or right of fixation. Rye position is indicated by the red dot. **B.** After a variable period (500–1500 ms), the fixation point disappeared. **C.** Upon fixation point offset, the monkey saccaded to the aperture. **D.** If the saccade occurred within 300 ms of fixation offset, reward was delivered 200 ms after the end of the saccade, during which time the eye movements are recorded.

Stimuli were presented on a computer monitor (Sony Trinitron Multiscan, 640×480 resolution, 120 Hz) placed 57 cm from the monkey. Eye position was monitored via infrared eye tracking (ISCAN Model ETL-400, 240 Hz). Experimental control was maintained with Cortex software (http://www.cortex.salk.edu). The monkeys had first to orient their gaze within 0.75 degrees of a 0.25 degree fixation point (Figure 1). After maintaining fixation for 200 ms, an aperture with one or two translating dot fields (dot fields: 2.75 deg radius, density: 5 dots/deg^2^, dot size: 0.05 deg, speed: 5.75 deg/s, direction: left or right) appeared in either the lower left or lower right visual field (7 deg eccentricity). After a variable period of time (500–1500 ms) the fixation point disappeared, signaling a saccade to the aperture. The monkey had to saccade to the aperture within 450 ms to receive a juice reward. The reward was delayed for 200 ms post-saccade. Eye movements during this 200 ms period were analyzed. The contrast of the dots that gave rise to motion was increased by increasing dot luminance, while holding background luminance at a constant value (5.32 cd/m^2^) throughout the display. This manipulation also resulted in an increase in the average mean luminance across the stationary aperture. Contrasts were computed using a standard root-mean-square contrast metric [6]. Each level of contrast was converted into a percentage by dividing by the RMS-contrast of a surface comprised of maximal brightness dots (194 cd/m^2^). This provided a logarithmic scale as is used in studies of contrast.

### Experiment 1 – Single Pattern

In Experiment 1, a single pattern of dots translated to either the right or left. The pattern was delimited by an invisible circular aperture: when dots moved beyond the boundary of the aperture, they were deleted and replaced by new dots appearing on the other side of the aperture. The contrast of the dots varied across trials (0.02% to 100% RMS-contrast). The contrast patterns were pseudorandomly interleaved across trials.

### Experiment 2 – Two Superimposed Patterns

In Experiment 2, two superimposed patterns of dots appeared in the aperture, translating in opposite directions (left, right). When these stimuli are presented to human participants, they are perceptually segmented into 2 surfaces moving in different directions [7], [8]. Similarly, monkeys can report the direction of 1 of 2 superimposed surfaces when segmented by color [9]. Thus the superimposed patterns of dots are seen as two transparent surfaces. In an individual session, one pattern was held at a constant low contrast, while the other pattern varied from that low contrast up to 100% RMS-contrast. The contrast values were pseudorandomly interleaved across trials. Across sessions the low contrast pattern varied from 0.02% – 0.9% RMS-contrast. As a control, we also included a condition where both patterns were 100% RMS-contrast.

### Experiment 3 – Changing Contrasts during the Saccade

In Experiment 3, two patterns of dots appeared in the aperture. In the Standard condition, one pattern was presented at 100% RMS-contrast and the opposing pattern was presented at 0.2% RMS-contrast. In the Equalized condition, the patterns first appeared at 100% and 0.2% RMS-contrast. At saccade onset, the contrast of the lower contrast pattern increased to 100%, thereby equalizing the contrasts of the two patterns. In the Unequalized condition, the two patterns began at 100% RMS-contrast and at saccade onset, one pattern was reduced in contrast to 0.2%. In the Reversal condition, one pattern began at 100% contrast and the other began at 0.2% RMS-contrast. Upon saccade onset, the contrasts of the two patterns were reversed. In the Presaccadic condition, the two patterns began at 100% and 0.2% RMS-contrast, but 100 ms prior to the disappearance of the fixation point (the go signal), the lower contrast pattern increased to 100% RMS-contrast. This is similar to the Equalized condition, except that the equalization occurred 100 ms prior to the go signal instead of at saccade onset.

### Eye Movement Analysis

Eye position estimates were collected at 200 Hz. Saccades were defined as eye movements whose mean velocity exceeded 20 deg/s, peak velocity exceeded 50 deg/s, and whose duration lasted at least 20 ms. Trials were excluded from analysis if a double-step saccade occurred in saccading to the aperture. Trials were also excluded if, after saccading to the aperture, another saccade or an eye blink occurred during the 200 ms analysis period. For each trial, the saccade detected by algorithm was also visually inspected to confirm accuracy. We collapsed across leftward and rightward motion and across left and right hemifields. Gain was computed as the average horizontal velocity over the 200 ms analysis window, divided by stimulus velocity. If the smooth eye movements followed the motion of the pattern perfectly, gain would be 1. Statistical tests were performed on gain.

### Latency Analysis

We computed the latency of pursuit onset across the population of eye position traces for all the trials in a given condition. We slid, by 5 ms increments, a 20 ms analysis window, testing for a significant difference (t-test) in eye position compared to eye position at saccade offset (baseline). The latency was defined to be the first time at which a significant difference was found that was maintained for 50 ms.

### Difference Analysis

In Experiment 3, we computed the time at which pursuit differed significantly between the Standard condition and each of the other conditions (Equalized, Unequalized, Reversal, Presaccadic). For each animal, we slid a 5 ms analysis window by 5 ms increments, testing for a significant difference (t-test) between the distributions of horizontal eye positions in the trials between each experimental condition and the Standard condition. The time of divergence was defined as the first 5 ms analysis window for which a significant difference was found that was maintained for the subsequent 50 ms.

## Results

### Experiment 1 – What is the effect of contrast on smooth eye movements?

In Experiment 1, we characterized the gain of smooth eye movements following a saccade to an aperture containing a moving field of dots, as a function of root-mean-square (RMS) luminance contrast [6]. Two monkeys performed the task illustrated in Figure 1. Upon fixation on a central point, a pattern of dots appeared within an invisible aperture in the lower right or lower left quadrant of the visual field. The field of dots moved either to the right or left. The contrast of the dots varied across trials. After a random period of time, the fixation point was removed, signaling the animal to saccade to the aperture. The animal was rewarded for saccading to the aperture within 450 ms of fixation point offset. Delivery of juice reward was delayed for 200 ms after the saccade ended. Smooth eye movements during this 200 ms post-saccadic window were analyzed.

**Figure 2:**
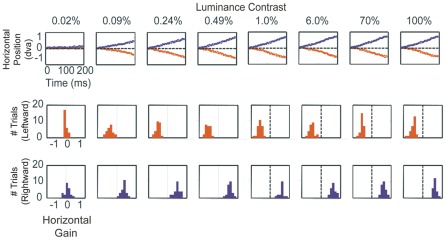
Responses to a Single Pattern of Varying Contrast. The top row of panels depicts average eye position over 200 ms starting at the end of the saccade for Monkey A, for 8 different contrasts, when the aperture was in the left hemifield. The blue lines are eye movement traces averaged across all trials on which the pattern moved to the right, while the red lines are average traces for leftward stimuli. The 2 rows of panels below show the distributions of horizontal gain of each individual trial, at each level of contrast for rightward stimulus motion (red histograms) and leftward stimulus motion (blue histograms). The point of gaze followed the motion of the pattern for all but the lowest contrast (for each contrast of 0.09% – 100%: two-tailed t-test, p<0.001; 0.02%: two-tailed t-test, p>0.05, n.s.).

Illustrative eye movements for one animal are shown in Figure 2. When the contrast was very low (0.02% RMS-contrast), the eyes did not pursue the target. This is illustrated for Monkey A in the top left panel of Figure 2, which shows average eye position traces computed across all trials in which a 0.02% contrast stimulus moved to the left (red trace) or right (blue trace). Oculomotor gain was computed each trial by dividing mean eye velocity over the 200 millisecond period, by stimulus velocity. At this level of contrast, the animal was able to see the stimulus, as indicated by its ability to accurately saccade to the aperture. However, at 0.02% contrast, the pattern was below the threshold for triggering a measurable post-saccadic smooth eye movement, as indicated by the overlapping distributions of oculomotor gain, appearing in the two panels below the 0.02% contrast eye trace for leftward trials (red) and rightward trials (blue). The two distributions are not significantly different from one another, according to a two-tailed t-test (p>0.05). It should be noted that though it was beyond our measurement resolution, it is possible the 0.02% contrast surface elicited a very slight degree of pursuit. As the contrast of the pattern increased, the gain of the smooth eye movements increased, as indicated by the separation of the eye position traces (top row) and the separation of the trial by trial gain distributions (Figure 2, columns 2–8).

**Figure 3:**
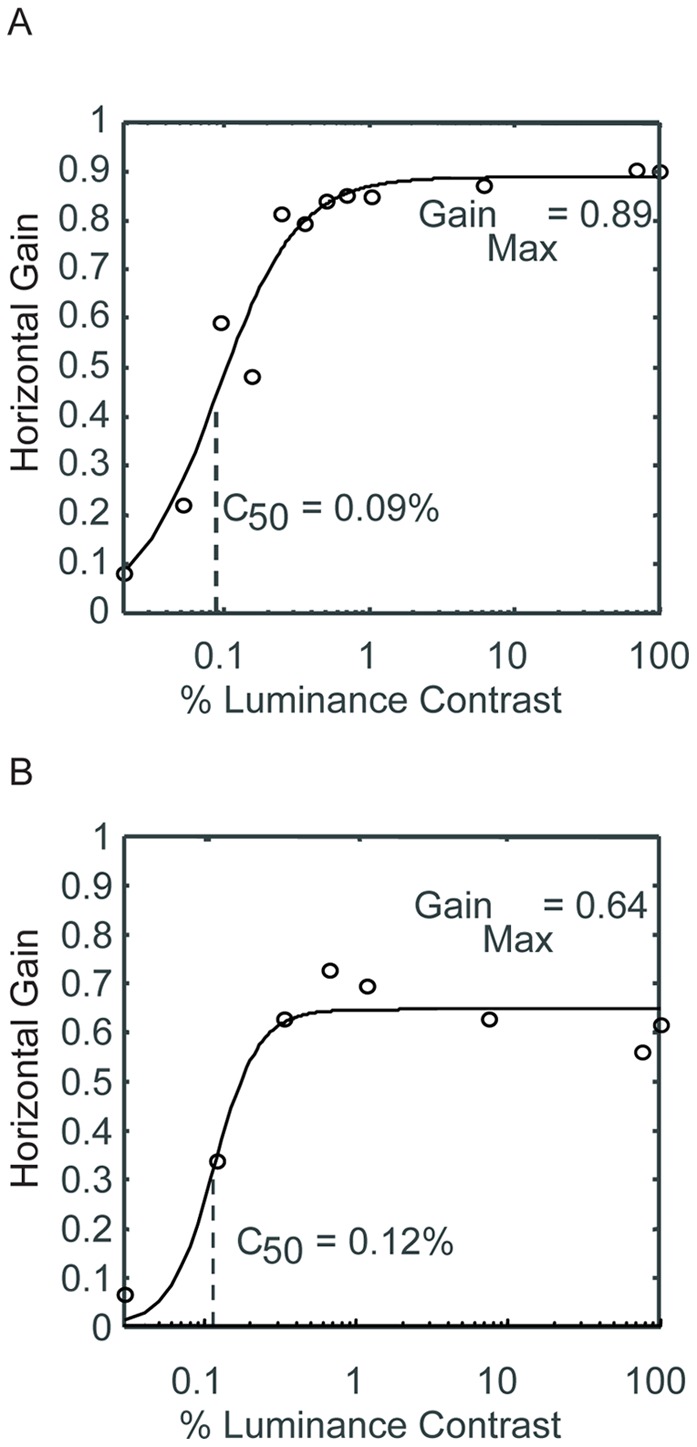
Contrast-dependent Oculomotor Gain Function. Oculomotor gain is plotted as a function of contrast. The data is fitted to the function: Gain  =  Gain_Max_ * X^n^/(X^n^ + C_50_^n^), where Gain_Max_ is the maximum estimated gain and C_50_ is the contrast that results in 50% of the maximum gain. **A.** The contrast-dependent oculomotor gain function (COGF) is plotted for Monkey A. In this subject, the maximum gain was 89% of the dot velocity, and the C_50_ point was 0.09% RMS-contrast. **B.** For Monkey B, the maximum gain was 64% of the dot velocity, and the C_50_ point was 0.12% RMS-contrast.

Figure 3 plots the average gain against luminance contrast for each animal (Monkey A, panel A; Monkey B, panel B). We fit the contrast-dependent oculomotor gain function (COGF), i.e., the relationship between luminance contrast and gain, with a function of the form:
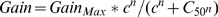
where *c* is the contrast of the dot field, *Gain_Max_* is the upper asymptote of the gain function, and C_50_ is the contrast that results in 50% of the maximum gain. For both animals, the gain saturated at less than 3% RMS-contrast, though the maximum gain differed across animals (Gain: Monkey A, 0.89; Monkey B, 0.64). The C_50_ points were similar (Monkey A, 0.09%; Monkey B, 0.12%).

We then determined the latency of pursuit onset for each of the contrasts in each animal. We could not compute latencies for the lowest contrasts as there was little to no pursuit (See Figure 2, leftmost column, 0.02% RMS-contrast). For the contrasts that did yield pursuit, there was no relationship between contrast and latency (Monkey A, range 15–35 ms, mean 22 +/− 5 ms, R^2^ = 0.015, p = 0.72, n.s.; Monkey B, range 20–35 ms, mean 26 +/– 6 ms, R^2^ = 0.007, p = 0.86, n.s.). In this study, we estimated pursuit onset based on position. Alternative latency estimates, including those based on velocity and those that correct for biases associated with the width of the estimation window might differ slightly, in absolute terms, from our estimate, but this would not be expected to materially change our conclusions, all of which were based on comparisons across conditions. Thus, the velocity of pursuit was modulated by contrast, but the initiation of pursuit was not.

### Experiment 2: How is the COGF affected by the addition of a second superimposed dot field moving in the opposite direction?

In Experiment 2, we asked how the COGF would change when we superimposed a second dot field moving in the opposite direction. The monkeys performed the same saccade task as in Experiment 1, except that two oppositely moving patterns of dots appeared within the aperture. The contrast of the added pattern was varied at random across a range of contrasts from 0% (no opposing motion) to 0.9% RMS-contrast, resulting in five COGF functions.

**Figure 4:**
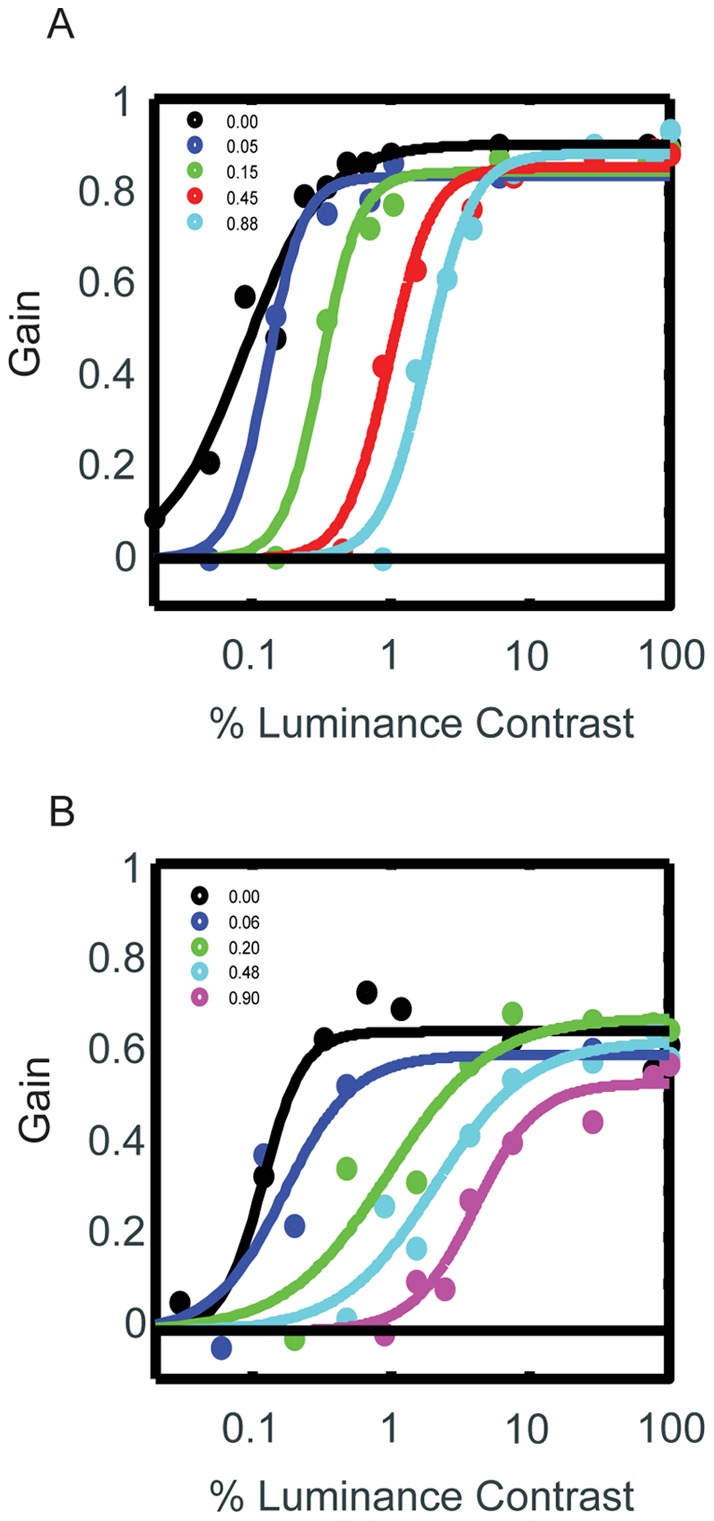
Effect of Opposing Contrast on COGF. Oculomotor gain is plotted as a function of contrast of the primary pattern. The contrast of the opposing pattern is depicted by each of the colors. Black represents no opposing pattern, i.e., when the contrast of that pattern was 0% RMS-contrast. **A.** This panel depicts the effect of the opposing pattern's contrast, in Monkey A. As the contrast of the opposing pattern increased, the COGF shifted further to the right. **B.** The same change with increasing contrasts of the opposing pattern held in Monkey B.

**Figure 5:**
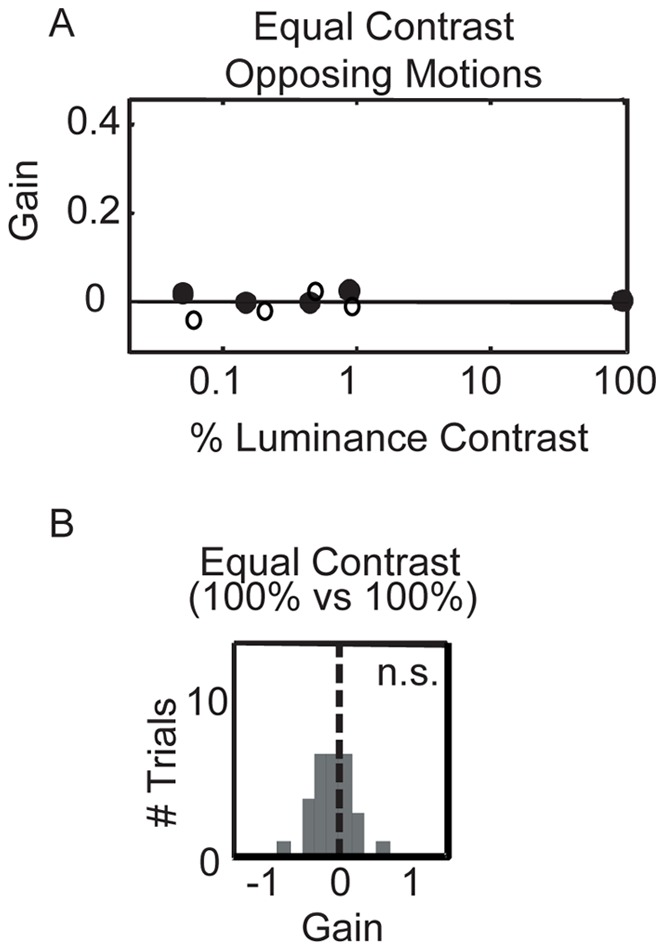
Oculomotor gain when opposing patterns are equal in contrast. A. The mean gain of the equal contrast conditions from Experiment 2 for Monkey A are plotted (filled circles) and Monkey B (open circles). Regardless of the actual contrast, at equal contrast, oculomotor gain is approximately zero. **B.** The distribution of gains resulting from opposing patterns both with 100% RMS-contrast (also plotted in panel A). The distribution is unimodal and is not significantly different from 0 (two-tailed t-test, p>0.05).

The results of Experiment 2 are shown in Figure 4. The black curves show the COGFs in the two animals when the contrast of the opposing pattern was zero (single pattern COGFs, replotted from Experiment 1). In both animals, the main effect of adding the opposing pattern was a rightward shift in the COGF whose magnitude increased with the contrast of the opposing motion. This shift was large enough to nullify eye movements when the two oppositely moving patterns were equal in contrast. Across the range of contrasts tested (0.05% – 100%), equating the luminance of the two patterns nullified the smooth eye movements (Figure 5A).

We next asked whether this shift resulted from a fraction of trials on which the visual system selected the opposite motion, as would be indicated by a bimodal distribution of gains. In order to maximize our ability to detect a bimodal distribution, we recorded an additional set of eye traces using opposite motions at maximum contrast. The distribution of smooth eye movements was unimodal (Figure 5B) and was not significantly different than zero (two-tailed t-test, p>0.05). Thus, we find no evidence that the visual system selects one of the two patterns (a winner-take-all selection) in the absence of a task demand to pursue a specific surface. Rather, eye movements appear to follow a unimodal velocity distribution whose mean falls between the velocities of the two patterns. This is consistent with the vector averaging of direction that has previously been reported for smooth pursuit using targets equated in contrast [1], [2].

### Experiment 3: When does the contrast difference influence smooth eye movements?

**Figure 6:**
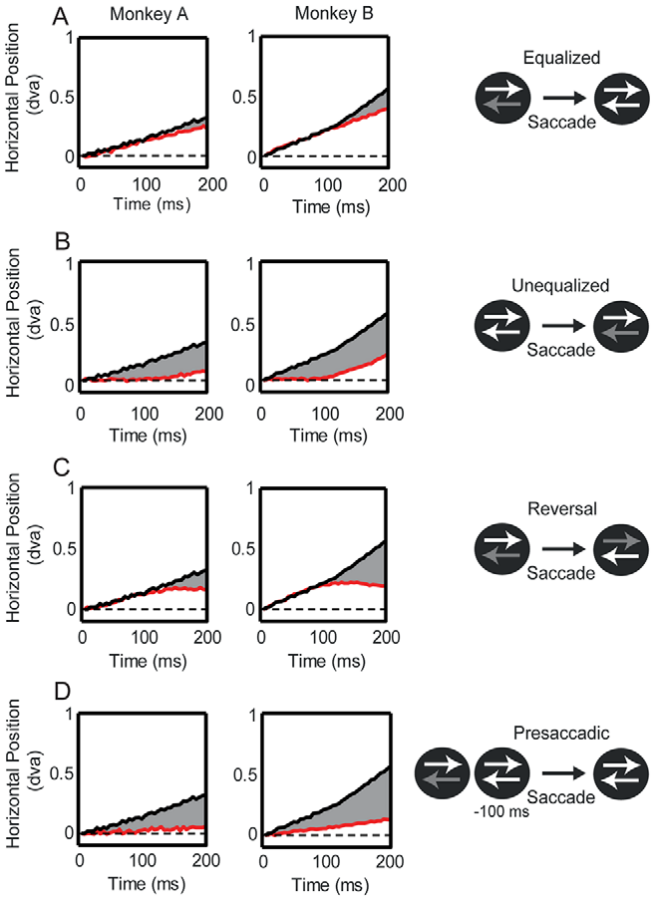
Quantifying the timing of smooth eye movement planning. The figure depicts the eye movements of Monkey A (left column) and Monkey B (middle column) over 200 ms starting at the end of the saccade. The black line in each panel shows the average eye position relative to saccade endpoint in the Standard condition, in which two patterns were present (0.2% and 100% RMS-contrast) throughout the trial. The red line in each panel shows the average eye position under the stimulus manipulation that was applied in that condition (depicted in the right column). Shading indicates the time points at which there was a significant deviation from the Standard condition. **A.** In the Equalized contrast condition (red line), unequal contrasts were equalized during the saccade. The effect of the initial contrast difference persisted for approximately 150 ms (Monkey A: 165 ms; Monkey B: 130 ms). **B.** In the Unequalized contrast condition, the two patterns began at 100% RMS-contrast and at saccade onset, one pattern was decreased to 0.2% RMS-contrast. The effect of differentiating contrast took 130 ms to emerge (Monkey A: 150 ms; Monkey B: 110 ms). **C.** In the Reversal condition, the two patterns began at 0.2% and 100% RMS-contrast, and contrasts were reversed upon saccade onset. Smooth eye movements followed the original higher contrast pattern for 130 ms (Monkey A: 140 ms; Monkey B: 120 ms) before the initial bias was weakened **D.** In the Presaccadic condition, the two patterns started out at 0.2% and 100% RMS-contrast, but 100 ms prior to removal of the fixation point, the lower contrast pattern increased to 100% RMS-contrast. While greatly diminished, smooth eye movements in the direction of the previously higher contrast pattern were still present.

In Experiment 3, we sought to determine when, in time, contrast differences affect smooth eye movements, by varying the time period over which the contrasts of the two patterns differed. For these experiments we used only a single contrast difference: 0.2% versus 100% RMS-contrast. In the “Standard” condition, the two patterns differed in contrast throughout the trial. As in Experiment 2, the eyes followed the higher contrast stimulus. This is shown by the upward sloping black line appearing in each panel of Figure 6. The black line is repeated in each panel, for comparison to each experimental manipulation, shown in red. In the “Equalized” condition (Figure 6A, red lines), the two patterns began unequal in contrast (0.2% and 100% RMS-contrast), but at saccade onset the lower contrast pattern increased to 100% RMS-contrast, thereby matching the other pattern. Upon landing within the aperture, the eyes initially moved in the direction of the initially higher contrast stimulus, regardless of whether or not contrast was equated during eye flight. The gain of the smooth eye movement began to significantly diverge from the Standard condition (Fig. 6A, black lines) at approximately 150 ms (Monkey A: 165 ms; Monkey B: 130 ms) after the end of the saccade (see Methods for algorithm to estimate time of divergence). Thus, the bias that was introduced by a contrast difference in the periphery during fixation was then maintained over the saccade and influenced eye movements after the contrast-equated stimuli were foveated at the end of the saccade for an additional period of approximately 150 ms.

On interdigitated trials, we introduced an “Unequalized” contrast condition, in which contrasts were initially equal, and remained so throughout fixation (100% and 100% RMS-contrast). Then, during the saccade, one of the patterns was reduced in contrast to 0.2%, setting up a contrast difference (0.2% and 100% RMS-contrast) that biases pursuit in favor of the higher contrast surface. If contrast-induced bias occurs only after the saccade, then this condition would be expected to produce the same gain as the Standard condition. Instead (Figure 6B, red lines), immediately after foveating the aperture, the eyes tracked neither pattern, despite this newly introduced large contrast difference. This contrast difference did not bias eye movements for the first 110–150 ms (Monkey A: 150 ms; Monkey B: 110 ms) following foveation of the aperture. This time frame is similar to that we observed in the Equalized condition, and the 150 ms latency previously reported for initiation of smooth pursuit eye movements in the presence of two oppositely moving, spatially separate targets [10].

The results of the Equalized and Unequalized conditions suggest that the initial phase of smooth pursuit is driven by contrast differences that occurred in the visual periphery, prior to the saccade, with relative contrasts of the stimuli foveated by the saccade exerting influence only after a delay of approximately 150 ms. Consistent with this, when we reversed the contrasts of the two patterns mid-saccade (Reversal, Fig. 6C, red lines), the bias resulting from the pre-saccade contrast difference (Fig. 6C, black lines) was still present for approximately 130 ms (Monkey A: 140 ms; Monkey B: 120 ms), before the reversal caused a significant divergence, relative to the Standard condition.

The results from the above experiments suggest that the contrast-dependent competition is resolved and the pursuit motor plan is formulated prior to the initiation of a saccade to the aperture. The pursuit motor plan is then automatically executed upon termination of the saccade. This leads to the very short latencies (<35 ms) for pursuit following the saccade, which is updated by post-saccadic stimulus conditions after a delay of approximately 150 ms. This value is consistent with visually evoked smooth pursuit to a target in the presence of an oppositely moving distractor [10].

Consistently across these conditions in Experiment 3, we find that post-saccadic eye movements are updated approximately 150 ms after the end of the saccade. While the eye movement plan can be updated by newly foveated motion, this is a somewhat time-consuming process, requiring on the order of 150 ms. In a final condition, we measured the time course over which pursuit planning can be influenced by differences in contrast occurring in peripheral vision, prior to the saccade. In this “Presaccadic” condition (Fig 6D, red lines), we presented unequal contrasts during fixation, and then equalized them 100 ms prior to the disappearance of the fixation point. The eye movement gain following the saccade was strongly reduced compared to the standard condition (Fig 6D, black lines), indicating that changes in the relative contrasts of peripheral motion can rapidly update pursuit eye movement planning. We do note that even though the two surfaces were equal in contrast prior to the saccade, the bias to pursue the previously higher contrast surface was not completely abolished (Fig 6D, red lines) producing small but significant pursuit at approximately 50 ms (Monkey A: 65 ms; Monkey B: 35 ms).

## Discussion

We find that smooth pursuit gain increases with the luminance contrast of the moving stimulus, but then saturates, with asymptotic gain differing across the two animals tested. This result is consistent with prior studies of the effects of varying contrast on speed perception [11], [12] and on smooth pursuit to a single moving target [13]. Importantly, the introduction of countervailing motion shifted this contrast-dependent oculomotor gain function (COGF) to the right, with shifts increasing with the contrast of countervailing motion. The rightward shift was sufficient to null out eye movements when the two patterns were equated in contrast. Countervailing motion required approximately 150 ms to influence post-saccadic eye movements. These results are consistent with earlier studies using spatially separate stimuli, which found that initial post-saccadic smooth eye movements can be characterized by vector averaging [1], [2]. The present experiments extend these findings, by characterizing smooth pursuit using spatially overlapping stimuli that varied in luminance contrast, and by examining the temporal dynamics of these changes on post-saccadic smooth pursuit eye movements. These results are also consistent with a recent study showing that global motion processing of a random-dot field is computed by weighting the motion energy of each individual dot by the relative contrast of that dot [14]. In the earlier study, a random-dot cinematogram was presented, and observers perceived global motion flow as biased in favor of the dots with the greatest luminance. The present study used very different stimuli: superimposed patterns of dots that moved rigidly, yielding the percept of two superimposed transparent surfaces. It also differs in that we measured smooth pursuit. Despite these differences, both studies are consistent in finding preferential processing of the most salient motion stimuli. Taken together, these studies suggest a common mechanism, operating at different stages of motion processing: the integration of individual elements into global motion [14] as well as motion transparency, as found in the present study.

One possible explanation for the observation that oculomotor gain increases before saturating at low contrast is that the oculomotor system may compute smooth pursuit on the basis of motion signals in areas where neuronal responses saturate at low levels of luminance contrast. Likely candidates are the middle temporal area (MT) and the medial superior temporal area [15]–[18]. Neurons in these areas are selective for the speed and direction of motion [19]–[21] and MT neurons exhibit contrast response functions that saturate at low luminance contrast [22]. Lesions of MT and MST lead to impairments in smooth pursuit [23], [24]. Stimulation of MT neurons can modulate the speed of pursuit and even induce smooth pursuit in the absence of stimulus motion [25].

One possible way in which the brain could combine the opposing contrast-dependent motion signals would be to additively combine the pursuit motions resulting from each stimulus. It would, however, also reduce the asymptotic movement at high contrast, which we did not observe. For example, as shown in Figure 3A, an opposing surface at 0.88% rms-contrast produced near asymptotic oculomotor gain. According to the additive model, the 0.88% contrast stimulus would null out the motion of an opposite 100% rms-contrast surface, which was not observed. Rather, as seen in Figure 4A the 0.88% surface had no effect on pursuit eye movement driven by a 100% contrast stimulus (asymptote, cyan line, compare asymptote, black line).

A relatively simple alternative model is a normalization model. Normalization models yield saturating contrast response functions. Increasing the divisive inhibition term causes a rightward shift in the contrast response function [26]. Thus, if we posit that the introduction of countervailing motion divisively inhibits the activity of neurons selective for the primary motion stimulus, this would induce a rightward shift in the contrast response functions of neurons tuned for the primary motion direction. This proposal seems plausible, given that normalization models have been used to fit responses of motion selective neurons in area MT [22], [27]. This class of models dates back to early modeling work of Sperling and Sondhi [28] and Grossberg [29] and have been extended to account for a variety of response patterns in neocortical neurons [22], [30]–[32], including different forms of attentional modulation [26], [33]–[35]. The model posits that elevation of luminance contrast increases the excitatory drive to a neuron and also activates increasing levels of inhibition resulting in a saturating contrast response function. The introduction of a null stimulus activates additional inhibition, resulting in a rightward shift in the neuronal contrast response function, which increases with elevations of the null stimulus contrast [32]. Thus, the present finding, showing that the saturating COGF shifts to the right with elevation of countervailing motion, is consistent with the proposal that motion signals provided to the oculomotor system for smooth pursuit undergo a normalization computation, possibly within areas MT and MST.

This proposal is also consistent with earlier studies of smooth pursuit eye movements that found when two moving targets appear within the visual field, the early phase of an ensuing pursuit eye movement is well described as a weighted average of the motions of the two stimuli [1], [2]. Neurophysiological studies in MT [22], [27] and V4 [33], [36] have found that neuronal responses to pairs of stimuli can be fit with a weighted average of the responses evoked by the two stimuli when each is presented alone. The normalization model has been able to fit this neurophysiological response pattern [26], [27], [33], [34]. Therefore, if we assume that MT neurons provide signals used to compute smooth pursuit, the normalization model offers a way to link our results with these earlier neurophysiological and oculomotor studies.

### Selection for saccades and pursuit

As we maneuver through the world, multiple objects move across our retinas in different directions and at different speeds. It would be wasteful for the pursuit system to plan a pursuit eye movement for every potential moving target in the field of view. Indeed, smooth pursuit normally occurs after a saccade has foveated a target, and it stands to reason that smooth pursuit eye movements should therefore be gated by the saccadic system. Earlier studies have suggested that selection of targets for saccades and pursuit do occur serially [37]. Pursuit eye movements involve automatically maintaining the target on the foveal region of the retina by minimizing retinal slip [38]. This computation is thought to be reliant on motion processing in areas MT and MST, which are connected to pursuit-related regions of the cerebellum [15]–[18]. In contrast, the saccadic system involves a network of frontal, parietal, and subcortical areas in addition to the cerebellum and brainstem nuclei [39], [40]. However, there is also evidence that pursuit and saccades share some neural mechanisms in common [41]–[43] and that the signals involved in covert preparation for a saccade also mediate selection for pursuit [44], [45].

The results from the present study provide support for partial functional overlap between theses two forms of selection. One difference between them was their luminance contrast thresholds. Saccades were made to single targets presented at contrasts that fell below the contrast required to drive pursuit. This suggests that the saccadic control system includes neural elements capable of detecting saccade targets that are too faint to drive selection for pursuit. Thus, the contrast sensitivity for direction discrimination may be different than that for spatial localization. However, while saccadic selection was a requirement in our task, pursuit was not. Our subjects might have had the capacity to engage pursuit at lower levels of contrast, if this were required by the task. Still, the difference in contrast thresholds between saccade and pursuit target selection suggests that the two systems may be partially separate.

One particularly interesting aspect of the present findings is that contrast differences introduced during fixation, when the saccadic target was in the periphery, biased smooth pursuit eye movements occurring after the target was foveated. This occurred even when the contrast difference disappeared while the eye was in flight. Therefore, selection for pursuit can be biased by events occurring prior to the saccade itself. For the pursuit to be based on pre-saccadic information, it could be programmed in parallel with saccade programming [45]. However, it is worth noting that fixation was maintained during the period between introduction of the saccade target (the two superimposed stimuli) and the disappearance of the fixation point. As the monkeys likely planned the saccade in advance of fixation offset, it is quite plausible that covert saccade planning may have gated selection for pursuit. The delay period between saccade planning and saccade initiation was likely longer than required for pursuit planning. Sequential motor plans for complex actions have already been found in the reach system and are maintained during a delay period [46]. Additional experiments, in which the saccade target is unknown in advance, would be needed to test this hypothesis regarding the saccade and pursuit oculomotor systems. It is worth noting that when the contrasts changed during the saccade, pursuit based on the new contrasts only occurred after 150 ms. This pursuit latency is consistent with prior research when initiating pursuit to one of 2 spatially separate targets [10]. Thus, 150 ms is plausibly the time required for the visual processing, pursuit planning and initiation of the resultant smooth pursuit eye movement, with visual processing requiring processing multiple stimuli and weighting according to relative contrast.

### Related studies of pursuit eye movements

In the present experiments, eye movements were biased by differences in luminance contrast. Recent work in human observers [47] suggests that other factors, such as differences in color, induce similar biases. Observers viewed superimposed patterns of dots similar to those used in Experiment 1, but color was varied instead of contrast. Participants saccaded to the superimposed patterns of dots and then pursued one of the surfaces, without being required to by task demands. Pursuit target selection was biased according to a color hierarchy: a red surface was pursued when paired with a green, yellow or blue surface. A green surface was pursued when paired with a yellow or blue surface. And a yellow surface was pursued when paired with a blue surface. Thus, selecting a pursuit target was biased for red > green > yellow > blue, even though all the surfaces were isoluminant, Pursuit target speed was also modulated, in this case based upon the distance between the two colors in color space. In the current study, the higher contrast surface was automatically selected for pursuit, and the oculomotor gain of that pursuit was determined by rightward shifts in the contrast-dependent oculomotor gain function. Therefore, bottom-up feature differences, such as color and contrast, between two superimposed moving surfaces automatically bias pursuit target selection and oculomotor gain, in humans and non-human primates, alike.

A recent study [48] examined the effects of varying contrast on the initiation of pursuit eye movements to two superimposed surfaces moving in orthogonal directions to one another. Consistent with prior studies, they found that when the two were equal in contrast, pursuit approximated a vector average response. However, when there was an 8-fold contrast difference, pursuit became winner-take-all, following the higher contrast surface. The authors suggested that this computation could be subserved by a normalization circuit. Our results are consistent with this proposal, but differ in several ways. Most importantly, we parametrically investigated contrast-dependence at multiple opposing contrasts, yielding a complete oculomotor gain function. Consistent with a normalization computation, we find that this curve is sigmoidal. Further, by reproducing the complete curve across a range of opposing motions, we were able to show that increasing countervailing motion causes a rightward shift without altering the upper asymptote, as expected from a normalization circuit that incorporates divisive normalization. In addition, by parametrically varying the timing of contrast variation, we were able to put temporal constraints on the mechanisms that mediate this effect.

Ferrera and Lisberger [10] found that pursuit latency for a moving target (∼100 ms) can be delayed (to ∼150 ms) when a second stimulus is presented moving in the opposite direction. They interpreted this delay in latency as reflecting the time required for visual search to select the target, prior to pursuit. In the present experiment, we found no difference in the latency of pursuit when we added the second oppositely moving stimulus. For single stimuli or pairs, latencies were similar (<35 ms). This might reflect differences in the stimuli: isolated moving targets in the earlier study versus superimposed patterns of dots in the present study. However, a more likely explanation is that in the present study the stimuli were present for 500–1500 ms before the fixation point was extinguished. Thus the monkeys could plan pursuit prior to saccade onset. When the contrast changed during the saccade, updating the pursuit plan necessarily occurred after the saccade completed, a condition comparable to Ferrera and Lisberger [10]. As shown in Experiment 3 (Figure 6) contrast changes during the saccade were reflected in pursuit eye movements 150 ms post-saccade, consistent with the 150ms latency reported by Ferrera and Lisberger [10].

### Comparison with the Ocular Following Response

An important issue to consider is the possible contribution of the ocular following response (OFR) to the smooth eye movements observed in this study. As opposed to pursuit eye movements, which serve to maintain gaze on a moving target, the OFR is a reflexive, short-latency smooth eye movement that serves to stabilize gaze, relative to the background, and likely involves different underlying neural mechanisms [49]–[51]. In the present experiments, two dot patterns moved in opposite directions. When the higher contrast surface was selected for pursuit, the reduction in oculomotor gain caused by the opposing surface may have resulted from competition within the pursuit system, as we have argued. If the opposing surface activated the OFR, it may also have contributed to the reduction in gain. However, the OFR is typically induced by background stimuli that cover a large region (>20 deg), or a full field texture. The aperture in the present experiments was considerably smaller at 5.5 deg. It would therefore be expected to have a minimal effect. In addition, OFR is typically generated by sudden and short (∼100 ms) translations [51]. The stimuli in this study were presented for 500–1500 ms during fixation, before the onset of the eye movement. Any OFR produced by the sudden onset of motion would be expected to diminish or disappear by the time pursuit occurs. Thus, even if the OFR contributed to the reduction in oculomotor gain induced by the addition of the opposed motion stimulus, it seems unlikely that it was a major factor.

### Conclusion

These results extend the weighted average model of smooth pursuit by characterizing how the weighting factors vary as a function of the contrasts of the two stimuli. Oculomotor gains saturated at relatively low contrast, consistent with the proposal that motion signals in MT, whose neurons saturate at low luminance contrast, drive pursuit. Adding a surface with countervailing motion shifted the contrast-dependent oculomotor gain function to the right. We suggest that this bottom-up selection via contrast may be mediated by a divisive normalization circuit, possibly operating in motion selective areas MT and MST.
